# Retrotransposition of gene transcripts leads to structural variation in mammalian genomes

**DOI:** 10.1186/gb-2013-14-3-r22

**Published:** 2013-03-13

**Authors:** Adam D Ewing, Tracy J Ballinger, Dent Earl, Christopher C Harris, Li Ding, Richard K Wilson, David Haussler

**Affiliations:** 1 Center for Biomolecular Science and Engineering, University of California, Santa Cruz, 1156 High Street, Santa Cruz, CA 95064, USA; 2 Howard Hughes Medical Institute, University of California, Santa Cruz, 1156 High Street, Santa Cruz, CA 95064, USA; 3 Broad Institute of MIT and Harvard, 5 Cambridge Center, Cambridge, MA 02142, USA; 4 The Genome Institute, Washington University, 4444 Forest Park Avenue, St. Louis, MO 63108, USA

## Abstract

**Background:**

Retroposed processed gene transcripts are an important source of material for new gene formation on evolutionary timescales. Most prior work on gene retrocopy discovery compared copies in reference genome assemblies to their source genes. Here, we explore gene retrocopy insertion polymorphisms (GRIPs) that are present in the germlines of individual humans, mice, and chimpanzees, and we identify novel gene retrocopy insertions in cancerous somatic tissues that are absent from patient-matched non-cancer genomes.

**Results:**

Through analysis of whole-genome sequence data, we found evidence for 48 GRIPs in the genomes of one or more humans sequenced as part of the 1,000 Genomes Project and The Cancer Genome Atlas, but which were not in the human reference assembly. Similarly, we found evidence for 755 GRIPs at distinct locations in one or more of 17 inbred mouse strains but which were not in the mouse reference assembly, and 19 GRIPs across a cohort of 10 chimpanzee genomes, which were not in the chimpanzee reference genome assembly. Many of these insertions are new members of existing gene families whose source genes are highly and widely expressed, and the majority have detectable hallmarks of processed gene retrocopy formation. We estimate the rate of novel gene retrocopy insertions in humans and chimps at roughly one new gene retrocopy insertion for every 6,000 individuals.

**Conclusions:**

We find that gene retrocopy polymorphisms are a widespread phenomenon, present a multi-species analysis of these events, and provide a method for their ascertainment.

## Background

Mammalian genomes contain thousands of pseudogenes - stretches of DNA sequence with homology to functional genes. As an example, pseudogene.org documents 17,061 human pseudogenes in build 65, and 19,119 mouse pseudogenes in build 60 [1-3]. A recent, more stringent survey identified 14,112 pseudogenes in the human genome [4]. Pseudogenes originate through a variety of mechanisms including retrotransposition of processed mRNAs (processed pseudogenes), segmental duplication, and inactivating mutations. Processed pseudogenes are derived from spliced transcripts and they lack the intron-exon structure of their source gene [5].

Retrotransposition refers to the insertion of DNA sequences mediated by an RNA intermediate [6]. In humans, this process is carried out chiefly through the reverse-transcriptase [7] and endonuclease [8] functions of the LINE-1 ORF2 protein, with assistance from the ORF1 protein, which binds RNA [9] and functions as a chaperone [10]. In addition to mobilizing its own transcripts, LINE-1 mobilizes other transcripts including, but probably not limited to, Alu [11], SINE-VNTR-Alu [12] and processed pseudogenes [13]. The specific reverse-transcriptase responsible for processed pseudogene formation varies among species depending on the retroelement content in the genome. For example, in *S. cerevisiae*, processed pseudogenes are mobilized by Ty1 elements [14]. In this study we refer to retroposed gene transcripts as gene retrocopies to avoid confusion with the functional connotations of terms such as 'pseudogene' and 'retrogene' [15]. When used, 'pseudogene' (or retropseudogene) refers to a non-functional gene retrocopy while 'retrogene' refers to a gene retrocopy with intact activity.

A growing number of contemporary studies highlight the extent to which individuals differ in terms of inserted retrotransposon sequences [16], but there has not been significant study of how mammalian genomes differ from one another and from the reference assembly in a given population due to gene retrocopy insertions, although detection of the phenomenon has been discussed briefly [17,18]. Retrogene insertion polymorphisms have been described in a study of 37 *Drosophila melanogaster *inbred lines [19] based on the detection of intron presence/absence polymorphisms.

Pseudogenes affect genome function in several important ways. Although most gene retrocopies lack the 5' promoter and regulatory regions present at the site of origin [5], mobilization to another genomic location can put the retrocopy in a novel regulatory context that may allow it to be transcribed [20-22]. Transcription of certain gene retrocopies can be either widespread, specific to a tissue or cell type, or specific to particular tumors [23]. Transcribed gene retrocopies can regulate the source transcript through an antisense mechanism [24], are a source of siRNAs [25-28], can affect the stability of the source transcript [29], and can affect expression of the source gene by providing a molecular sponge that competes with the source transcript for miRNA binding due to sequence similarity to the source gene [30]. Retrocopies and retrogenes can exert direct effects if the nearby genomic architecture promotes their expression, as is the case for a novel insertion of the *FGF4 *transcript in the domestic dog, which leads to the chondrodysplastic phenotype that typifies many dog breeds [31]. On an evolutionary timescale, the process of gene duplication through retrotransposition of processed transcripts constitutes a major mechanism for new gene formation [32], typified by examples such as the *jingwei *gene in *Drosophila *[33].

Here, we refer to a processed gene transcript that is present as a retrotransposed insertion in one or more individuals but absent from the reference genome as a gene retrocopy insertion polymorphism (GRIP). Insertions that are not polymorphic and not transmissible (somatic insertions) are not referred to as GRIPs. We present evidence that the interspersed insertion of processed mRNAs into the genome is an ongoing mechanism of mutation in humans, mice, and chimps, and can occur in tumors. Additionally, the availability of our application for detecting these events will enable all large-scale genome sequencing projects to include gene retrocopy insertions in their analysis of genomic variation.

## Results and discussion

### A catalog of non-reference human gene retrocopy insertions

**Figure 1:**
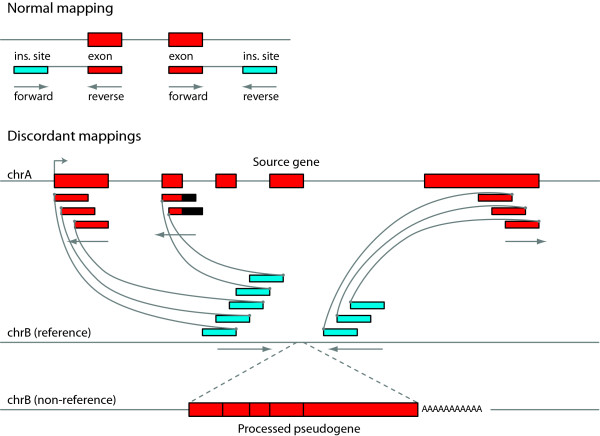
**Schematic overview of our method for detecting non-reference gene retrocopy insertions from paired read mappings**. Read pairs are represented by two boxes for the sequenced portion of the paired read, joined by a line representing the unsequenced region (not to scale). Reads aligning to exonic sequences are colored red, and boxes aligning to non-exonic sequences are colored blue. For genomic intervals with no significant structural changes relative to the reference, reads will map normally as depicted in the upper panel. Note the forward-reverse orientation pattern of the read pair mappings as indicated under the sequenced ends. Non-reference gene retrocopy insertions (bottom panel) are represented by a series of discordant read mappings in a common interval (blue boxes) where one end of each read matches a distal exon on a common gene annotation (red boxes). The minimum interval between the left and right groups of blue boxes defines the start and end coordinates used in Additional file 1: Tables S2, S4-6, and S9. For Illumina paired reads, the forward-reverse sequencing scheme means that the non-exonic end of paired reads spanning the 5' junction is mapped in the forward orientation and the non-exonic read of the pair spanning the 3' junction is mapped in the reverse orientation (see arrows). Thus, the regions joined by oriented paired reads between reference chrB and the gene on reference chrA form a path that indicates a gene retrocopy insertion on the chrB allele in the individual genome from which the paired reads were derived. As depicted on the non-reference version of chrB, processed gene retrocopies lack introns, and the resulting exon-exon junctions are detectable by local assembly.

Since GRIPs are largely undescribed, we sought to establish a catalog of insertions detectable by our method using the data available through the 1,000 Genomes Project [34]. We downloaded the alignments for 939 low-pass genomes from 13 self-identified populations available from the February 2011 release; a full listing of genomes is available in Table S1 in Additional file 1. Since these genomes are sequenced at relatively low depth, we analyzed all samples together using the strategy illustrated in Figure 1 and described in the material and methods section (GRIPper). This allows us to call insertions shared between multiple individuals, but likely has lower sensitivity to detect insertions present in only one individual. In total, we describe 39 GRIPs present in one or more individuals in this set of samples (Table S2 in Additional file 1).

In addition to samples sequenced by the 1,000 Genomes Project, we took advantage of the many samples sequenced to high depth by The Cancer Genome Atlas (TCGA) [35]. One aim of TCGA is to study the whole genomes of tumor and normal samples obtained from the same patient. We analyzed 85 paired genomes sequenced to high coverage depth (Table S3 in Additional file 1) and found 26 distinct GRIPs (Table S4 in Additional file 1). This dataset also provided us with the opportunity to search for cancer-specific somatic gene retrocopy insertions.

**Figure 2:**
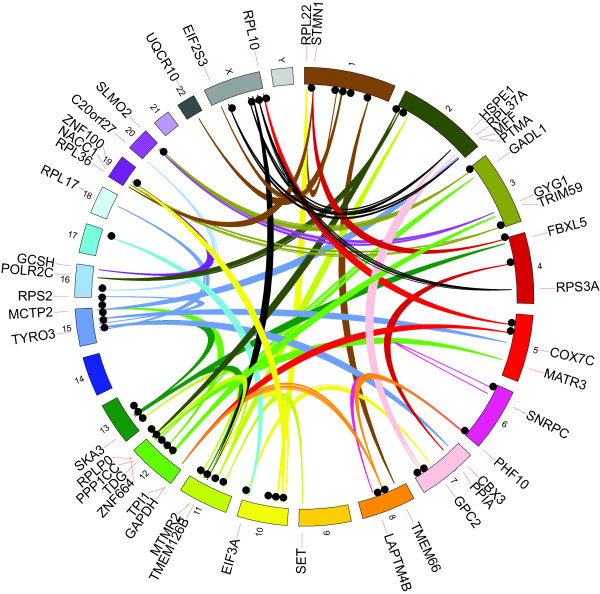
**Locations of 48 non-reference gene retrocopy insertion sites in the human genome based on reads mapped to source genes**. Discordant read mappings are represented by links colored based on the chromosome of the source gene. Insertion sites are represented by black circles, and the gene labels are based on the position of the source genes.

There was an overlap of 17 insertions between the two sets of genomes giving a total of 48 distinct gene GRIPs derived from 45 source genes (Figure 2, Table S5 in Additional file 1), since some genes produced mRNAs that were inserted into multiple distinct locations in one or more genomes. Of the 48, we could find breakpoints for 40 on at least one end, and found breakpoints for both the 5' and 3' junctions for 29 insertions. Of those 29, 28 had target site duplications typical of retrotransposed sequences, and one did not. Of these insertions, 21 out of the 48 are in introns and one has a breakpoint in an exon (a copy of *UQCR10 *inserted into exon 2 of *C1orf194*). Given the 45.75% genome-wide coverage of the gene annotation set used (see Materials and methods), this is not a significant enrichment of insertions occurring in annotated genes.

Most of these insertions bear the hallmarks of processed transcript insertions generated by retrotransposition. The insertion side of the 3' junctions terminates in poly-A sequences; we detected target site duplications in all instances where both junctions are detectable, and we could obtain exon-exon junctions from 39 out of 48 of the inserted sequences through a local sequence assembly approach (see Materials and methods and Additional file 2 for junctions derived from 1,000 Genomes samples). Predicted endonuclease cleavage sites agree with the consensus TTTT/AA reported in prior studies (Figure S5 in Additional file 3) [8,36].

In order to estimate the sensitivity and specificity of our detection scheme, we created a total of 2,000 simulated processed gene retrocopy insertions from 200 source genes and spiked them into the BAM file for sample TCGA-60-2711-11 and detected them by running GRIPper (see Materials and methods, Tables S10 and S11 in Additional file 1). The overall precision was 100% at the effective minimum read depth for paired TCGA samples (60× from the combined contribution of a tumor genome sequenced to 30× and the matched normal genome sequenced to 30× depth) and recall was 75.1% (Table S10 in Additional file 1). We found that recall varies depending on the identity of the source gene (Table S11 in Additional file 1, Figure S4 in Additional file 3).

### Functional characteristics of source genes

**Figure 3:**
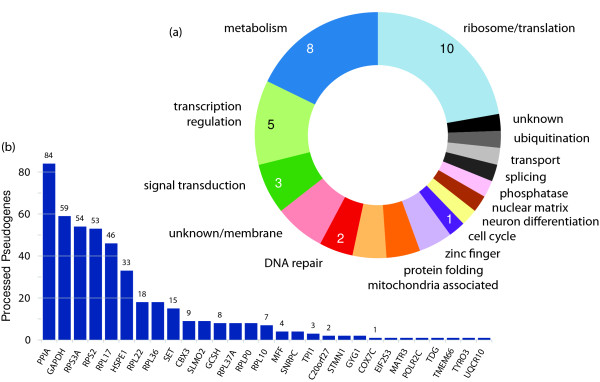
**Gene retrocopy insertion annotations**. **(a) **Functional classification of retrocopy source genes based on gene ontology and manual curation. The genes associated with each functional classification can be found in Table S8 in Additional file 1. **(b) **Number of annotated processed pseudogenes in the human genome reference assembly (GRCh37) (y-axis) for each source gene associated with a gene retrocopy in this study (x-axis). Processed pseudogene annotations were derived from pseudogene.org human build 65 [1,3].

As expected, many of the source genes that contributed new insertions fall into the same functional categories as the source genes for pseudogenes that are already present in the reference genome sequence. Examples include genes encoding proteins associated with ribosomal function, and genes involved in metabolic processes, transcriptional regulation, and signal transduction (Figure 3a). By examining the enrichment for functional annotations through DAVID [37], we see that a number of gene ontology (GO) terms associated with ribosomal functions are strongly enriched in the set of source genes (Table 1). Many of the retrocopy source genes also have other copies present elsewhere in the reference (Figure 3b). These include highly pseudogenized genes like cyclophilin A (*PPIA*) and *GAPDH *[1], and this result is consistent with the general observation that genes expressed in a wide range of tissue types, particularly those highly expressed in germ cells, are more likely to yield retrotransposed copies [38].

**Table 1:** GO term enrichment for human GRIP progenitor genes

GO term	*P*	Fold enrichment	FDR
GO:0006414: translational elongation	4.78 × 10^-9^	30.61	6.24 × 10^-6^
GO:0006412: translation	8.63 × 10^-8^	11.68	1.13 × 10^-4^
GO:0003735: structural constituent of ribosome	2.66 × 10^-7^	17.17	3.06 × 10^-4^
GO:0033279: ribosomal subunit	1.37 × 10^-6^	18.89	1.53 × 10^-3^
GO:0005840: ribosome	1.91 × 10^-6^	12.85	2.14 × 10^-3^
GO:0022626: cytosolic ribosome	2.92 × 10^-6^	25.59	3.28 × 10^-3^
GO:0005198: structural molecule activity	3.36 × 10^-5^	55.69	3.87 × 10^-2^
GO:0015934: large ribosomal subunit	3.58 × 10^-5^	25.78	4.02 × 10^-2^
GO:0044445: cytosolic part	6.23 × 10^-5^	13.64	7.01 × 10^-2^
GO:0030529: ribonucleoprotein complex	7.34 × 10^-5^	6.04	8.24 × 10^-2^

GO: gene ontology, FDR: False discovery rate

### Detection of cancer-specific gene retrocopy insertions

The genomes sequenced by TCGA include DNA derived from both normal tissue and a tumor sample taken from the same individual, enabling discovery of putative cancer-specific somatic variants. We analyzed pairs of tumor and normal genomes from 6 different types of cancer: 24 pairs from acute myeloid leukemia (AML) patients, 12 breast cancer (BRCA), 5 colorectal adenocarcinoma (COAD), 15 glioblastoma multiforme (GBM), 6 lung adenocarcinoma (LUAD), 13 lung squamous carcinoma (LUSC), and 10 ovarian carcinoma (OV). In screening these 85 pairs of tumor and normal genomes by combining the calls as described in the Materials and methods section, we discovered three novel somatic gene retrocopy insertions from two lung tumors with no corresponding read pairs in the matched normal samples and no supporting read pairs in any other sample in this study. The three source genes are selenoprotein T precursor (*SELT*), smooth muscle myosin heavy chain 11 (*MYH11*), and a spliced non-coding RNA known as *Homo sapiens *growth arrest-specific 5 (*GAS5*). While the presence of these genes is not enough to make any causative link with carcinogenesis in this patient, this does strongly suggest that somatic insertions of spliced mRNAs derived from protein-coding genes may occur, at least in the context of cancer. We note that *MYH11 *rearrangements involving *CBPβ *are implicated in cancers including acute myeloid leukemia [39] and sarcomas of the small bowel [40,41], and *GAS5 *depletion has been noted in breast cancer [42]. We note that the *MYH11 *insertion site occurs in a region that is sometimes deleted as a segregating variant cataloged in the Database of Genomic Variants [43], but the sample LUSC-2722, which has the novel *MYH11 *retrocopy in the tumor genome, does not have this deletion (Table S12 in Additional file 1). Somatic LINE-1 mediated retrotransposition events have been observed in lung, colon, ovarian, and prostate tumors for transposable element transcripts [44-46], but the mobilization of gene-derived transcripts is novel, and may be a means for the amplification of oncogene copy number in some tumors. The discordant read mappings leading to these three calls are shown in Figures S1 to S3 in Additional file 3.

### Novel gene retrocopy insertions in inbred mouse strains

We sought to extend our catalog of GRIPs to mice, as the deeply sequenced genomes of 17 different inbred mouse strains are now available [47], and a small set of GRIPs have been described [48]. We applied the same method as described for detecting GRIPs by substituting mouse genome annotations, and identified a total of 755 insertions from 610 distinct source genes (Table S6 in Additional file 1). We found that 63 loci overlap with structural variants obtained from a mouse of the DBA inbred strain using HYDRA-SV [48]. Since the mouse reference (mm9/NCBI m37) is assembled from sequences derived from the C57BL/6J strain, it is not surprising that we only detected one novel GRIP in that strain, which could have occurred in the generations between the last common ancestor of the mouse sequenced by the Mouse Genome Sequencing Consortium [49] and the more recently sequenced individual [47]. Of the 755 insertions identified in our analysis, 201 (26.62%) occurred in annotated genes. This is a significant depletion compared to the 40.38% of the genome covered using the UCSC Genes annotation set (see Materials and methods, *P *= 1.76 × 10^-14^, proportions test).

**Figure 4:**
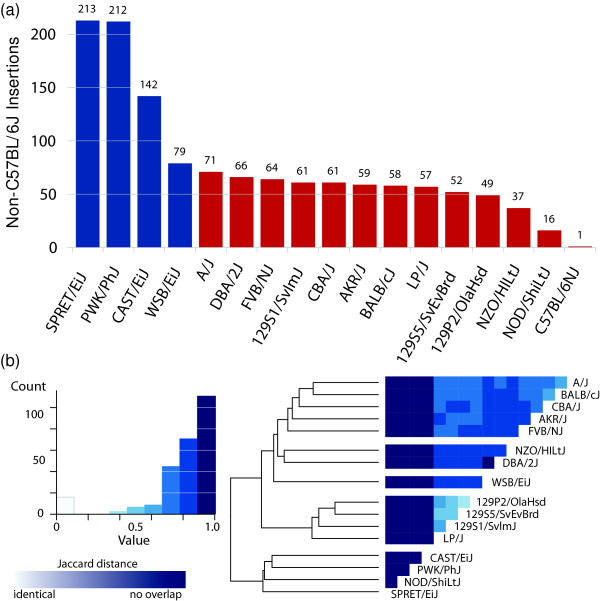
**Gene retrocopy insertions in mice**. **(a) **Number of gene retrocopies absent from the C57BL/6J reference (y-axis) present in each of 17 inbred mouse strains [47] (x-axis). **(b) **Heatmap created by the heatmap.2 function in the gplots package in R based on the Jaccard distance from pairwise comparison of GRIP alleles between strains (Materials and methods). C57BL/6NJ was left out of the inter-strain comparison of non-reference GRIPs because all but one insertion was shared with the C57BL/6J reference. As indicated on the histogram to the left of the heatmap, distances range from 0 (white, GRIP profile) to 1 (dark blue, no overlap in GRIP profiles). Hierarchical clustering of similarity indices generally recapitulates the breeding history of wild and inbred mouse strains [50].

The representatives from the 17 inbred strains differ from the C57BL/6J reference by a variable number of gene retrocopy insertions, generally correlating with what is known about the history of these strains [50] and in agreement with the degree to which transposable element polymorphisms are shared between strains [51]. All of the strains derived from *Mus musculus domesticus *(excluding C57BL/6J, which is also *M. m. domesticus*) have a mean of 56 GRIPs in their genomes that are not in the C57BL/6J reference (Figure 4a). In contrast, CAST/EiJ (*M. m. castaneus*), PWK/PhJ (*M. m. musculus*), and SPRET/EiJ (*M. spretus*) are strains derived from wild mice and have 213, 212, and 142 non-C57 gene retrocopies, respectively. WSB/EiJ is a wild-derived *M. m. domesticus *strain, and has the most non-C57 GRIPs of the *M. m. domesticus *strains sampled. Excluding C57BL/6J, any pair of the remaining 16 mouse inbred strains differ from one another by an average of 134 insertions. Excluding the four strains derived from wild mice, the remaining 12 lines differ from each other by an average of 68 insertions. The distance [52] between mouse strains in terms of shared GRIPs recapitulates the genetic ancestry of the strains to some degree, as might be expected (Figure 4b). For example, the 129 substrains are closely grouped together along with LP/J, which is closely related [50] (inbred stain genealogies chart available from Mouse Genome Informatics [53]).

### Novel gene retrocopy insertions in chimpanzees

In addition to whole-genome sequence data available for humans and mice, genome sequences for ten individual chimpanzees are available through the PanMap project [54]. These genomes were sequenced to approximately 10× average depth and are available in .bam format aligned to the Chimp Genome Sequencing Consortium 2.1/panTro2 reference assembly. We downloaded these and used the same pooling strategy used for the low-coverage data from the 1,000 Genomes Project to identify novel gene retrocopy insertions present in one or more of the ten individual chimps but absent from Clint, the reference chimp. In total, we identified 19 novel GRIPs, 9 of them in introns (Table S9 in Additional file 1).

### Distribution of GRIPs in human populations

**Figure 5:**
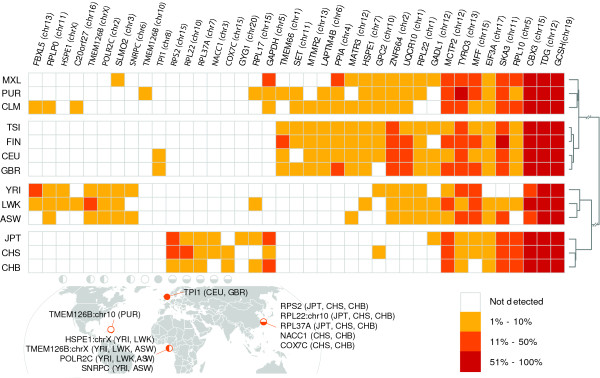
**Population distribution of human gene retrocopy insertions**. Rows represent self-described human populations with three-letter designations as used by the 1,000 Genomes Project. Columns represent 48 retrocopies. Open squares indicate the GRIP (row) was not detected in the population (column) and filled squares indicate that a GRIP was detected in the corresponding population at a frequency indicated by the color of the square. Hierarchical clustering of populations was performed using the Jaccard distance between each pair of insertion profiles. Population-specific GRIPs restricted to either single populations or groups with geographically similar ancestry are shown according to geographic locality. Correspondence between indicated geographic locations and columns representing allele frequencies is indicated by open, closed, or partially closed circles. ASW: African ancestry in south-west US; CEU: Utah residents with northern and western European ancestry; CHB: Han Chinese in Beijing, China; CHS: Han Chinese in southern China; CLM: Colombian in Medellin, Colombia; FIN: Finnish from Finland; GBR: British from England and Scotland; GRIP: gene retrocopy insertion polymorphism; JPT: Japanese in Tokyo, Japan; LWK: Luhya in Webuye, Kenya; MXL: Mexican ancestry in Los Angeles; PUR: Puerto Rican in Puerto Rico; TSI: Toscani in Italy; YRI: Yoruba in Ibadan, Nigeria

As with any heritable genomic polymorphism, GRIPs can be restricted to certain populations. The data from the 1,000 Genomes Project provide us with the opportunity to ascertain whether a given GRIP occurs more frequently in one population versus another. The population distribution for the 39 GRIPs from the 13 populations represented in the analyzed 1,000 Genomes Project data is shown in Figure 5. A number of these appear to be restricted to a particular geographical area of origin, such as insertions of *POLR2C*, *HSPE1*, and *SNRPC *mRNAs in individuals with self-reported African ancestry, and *COX7C*, *NACC1*, *RPL22*, *RPS2*, and *RPL37A *in individuals self-identified as belonging to Chinese or Japanese populations. The subset of the 1,000 Genomes Project data that we analyzed to obtain these insertions is low-coverage by design, to allow for detection of common alleles across a large number of individuals [34]. The cancer and normal pairs of deeply sequenced genomes from TCGA allow for the detection of more rare alleles, which is reflected in the five insertions found in only one TCGA individual versus only one insertion found in only one individual in the 1,000 Genomes low-coverage data (Table S5 in Additional file 1).

### Estimating the rate of gene retroposition in humans

The rate at which new gene retrocopies are formed by retrotransposition may be related to the rate of new gene formation. Our population-level data in humans allows a straightforward estimate similar in method to a previous estimate of retrotransposition for LINE-1 elements [55]. Watterson's equation [56] estimates the mutation rate *μ*, which in this context refers to the per generation rate of processed gene transcript retroposition (see Materials and methods). Using the 48 non-reference human GRIPs identified from the 1,024 human genomes analyzed in this study, we estimate that 1 in every 6,256 individuals has a novel, heritable, gene retrocopy. Since this ignores any segregating retrocopies in the reference genome, we sought to estimate the number of reference retrocopies by cross-referencing the deletion calls from the 1,000 Genomes Project [57] with annotated pseudogenes in the human reference genome. We found evidence for 10 GRIPs in the reference (see Materials and methods) yielding a total of 58 segregating insertions for 1,025 individuals (when the reference genome is included as one individual). This increases our estimate of *μ *to 1 new gene retrocopy insertion per 5,177 individuals per generation (see Materials and methods). In order to apply Watterson's formulae without bias, the chosen markers must be selectively neutral. A Tajima's D test yields a value of -0.99, indicating that while there may be some tendency toward purifying selection, the detected human GRIPs are, when considered on the whole, under neutral selection [58], validating this method of estimation. Performing the same estimation for chimpanzees using an effective population size of 11,413, which was calculated from the same whole genome sequence data [51], we arrive at an estimate of 1 new insertion per every 6,804 chimps, quite comparable to humans with the small discrepancy most likely due to a lack of information concerning pseudogene deletions relative to the chimp reference assembly.

## Conclusions

A large fraction of the human genome is covered by copy number variants (CNVs), including regions containing genes [59], and a number of recent publications have highlighted the extent of variability in gene copy number due to CNVs between individual humans. Starting from a large-scale set of deletions detected in human populations [60], Schrider and Hahn calculate that any two humans differ by over 100 gene-containing CNVs [61]. Approximately 9% of human genes appear to vary in copy number, mostly between 0 and 5 copies [62], likely through segmental duplication. The data we have presented here add to what is known about gene copy number variation by highlighting another mechanism separate from the large duplications that cause copy number variability of intron-containing gene loci. Through retrotransposition, GRIPs occur as interspersed insertions of processed transcripts. Whereas segmentally duplicated genes are likely to share the same regulatory regime, gene retrocopy insertions often mobilize copies into novel regulatory contexts, where they tend to experience an increased likelihood of adaptive evolution [63]. Many of these new gene retrocopy insertions will be inactive due to missing promoters, frameshifts, and truncation. That said, the subset of GRIPs that are recent enough not to be lost or fixed through genetic drift are likely to be more recent insertions and likely to have suffered fewer inactivating mutations to the open reading frame and any intact regulatory elements.

It is clear that processed gene transcripts are retrotransposed in the germline, and by extension one might imagine that this also occurs in somatic tissues. Transgenic mice with a LINE-1 cassette facilitating detection of insertion events show extensive variation in transposition frequency across tissues [64], and in particular, neural progenitor cells in the brain [65]. There is evidence for somatic retrotransposition during early development in *Drosophila *[66] and in humans [67]. Somatic retrotransposition of retroelements may also occur in human cancers [44,45] and contributes to a variety of human diseases [68]. We have demonstrated that insertions of retrotransposed processed transcripts can contribute to somatic variation in tumor tissue. Given this observation, studies of somatic retrotransposition of processed mRNAs in a variety of somatic tissues including the brain may yield novel retrocopy insertions, given evidence for elevated retrotransposition in some specific neural tissues from quantitative PCR [69] and targeted ascertainment of insertion sites [70]. That said, a recent study indicates some neural tissues do not appear to support a high level of retrotransposition [71].

Each new insertion of a gene retrocopy presents a new opportunity for the evolution of a new gene or the modification of an existing function at the site of insertion. There are a number of examples where inserted gene retrocopies have acquired new functions [20]. One notable example is the insertion of cyclophilin A (*PPIA*) into *TRIM5α *in the owl monkey leading to a novel gene fusion that confers resistance to HIV-1 infection [72,73]. A similar mutation involving the insertion of a cyclophilin A retrocopy into *TRIM5α *also occurred independently in rhesus macaques, leading to resistance to HIV-2 and feline immunodeficiency virus infection [74,75]. In total, we report 22 human, 201 mouse, and 9 chimp GRIPs in introns or exons that could lead to novel gene fusions with modified functions [21]. While human GRIPs occur in annotated genes about as often as would be expected by chance, we identified a marked depletion of mouse GRIPs in genes. This may indicate purifying selection due to deleterious effects on the genes hosting the GRIPs. In any case, this observation illustrates that the ability to detect this form of genomic variation opens new questions about the biological consequences of gene retrocopy insertion and provides a starting point for further investigation. In general, this study will provide a foundation for future investigation into the functional consequences of gene retrocopy insertion polymorphisms.

## Materials and methods

### Gene retrocopy insertion detection from mapped paired end reads

Paired end reads consist of two DNA sequences flanking an internal unsequenced region. Given the average insert size of a sequencing library, and the locations relative to a reference genome where either end of a paired end fragment map, a pair of mappings is termed concordant if the sequenced ends are mapped to the reference genome at an interval and orientation compatible with the library construction. Conversely, a pair of mappings is termed discordant if the paired ends are mapped too far apart or in the wrong orientation relative to the reference genome to which they are mapped. Given sufficient read depth and agreement between multiple paired reads, discordant read pairings can contain information about genome rearrangements relative to the reference if the rearrangements bring two pieces of the genome into proximity that are distant from one another in the reference genome. Here, we use discordant read mappings to detect GRIPs by finding multiple discordant mappings that connect exonic sequences to a consistent location distant from the exons. We refer to the genome or genomes from which a sequencing library was generated and analyzed as the query genome. For some region of a chromosome, if the sequence of the query genome matches the sequence of the reference genome, read pairs mapped to that region will be concordant as shown in the normal mapping of Figure 1. Alternately, if a region in the query genome contains a structural variant (insertion, deletion inversion, and so on) relative to the reference, some or all of the read pairs mapping to that location may be discordant. Figure 1 also demonstrates the pattern of discordant mappings indicative of a gene retrocopy insertion in the query genome. In order to confidently predict the presence of a gene retrocopy in a query genome or genomes, we require at least eight distinct mappings between the source gene and its insertion location, with at least two mappings spanning each junction. Illumina sequencing chemistry yields paired reads where the first read in the pair is sequenced on the top strand and the second read is sequenced on the bottom strand, such that the first read maps to the top (+) strand of the reference genome and the second read maps to the bottom (-) strand of the reference genome. Given this property, the reads mapping to the 5' side of the predicted insertion site must be on the top strand and the reads on the 3' side of the site must be on the bottom strand. Likewise, the mappings of the discordant reads themselves must be consistent with this pattern. We also require that the reads mapping to the source gene must correspond to at least two distinct exons. Additionally, we filter out putative insertion sites where the site is in a region of the genome that contains an annotated or unannotated pseudogene. Unannotated pseudogenes are ascertained by comparing the insertion site +/- 500 bp to the rest of the reference genome using BLAT [76]. This method (GRIPper) was implemented in Python using pysam [77] and is available from github [78]. An archival version of the software is also available as Additional file 4; however, we suggest using the most up-to-date version via github.

### Breakpoint ascertainment from soft-clipped reads

Many of the human samples analyzed in this study were mapped using bwa [79], which allows for part of a read to align as long as the seed sequence meets the minimum mismatch criteria. The unaligned portion of these mappings is marked as soft-clipped. This provides a convenient means to check for breakpoints by looking for consistent break ends corresponding to the 5' and 3' junctions of the inserted gene retrocopy. Target site duplications are ascertained by searching for correspondence between the sequences on either side of the breakpoint.

### Local sequence assembly to identify exon-exon junctions

In order to identify exon-exon junctions that are present in inserted processed gene retrocopy sequences, we employed a two-stage local assembly strategy. First, read pairs that map within 500 bp of a predicted insertion site that are discordant, one-end-anchored (reads where the mate is unmapped), or have at least one read in the pair that is soft-clipped are used as input to a short read assembler. For a first attempt at assembly, we use Velvet [80] with a *k*-mer size of 31, the shortPaired option to indicate the reads were paired, and an insert length of 300. The resulting contigs are aligned back to the reference genome using BLAT [76] to identify reads that map to exonic sequences corresponding to the source gene and without aligning to the intervening introns (spliced alignments). The majority of junctions are ascertained in this first step using Velvet which utilizes de Bruijn graphs to guide assembly. Secondarily, the discordant, one-end-anchored, and soft-clipped reads corresponding to the remaining insertions for which an exon-exon junction was not apparent were then assembled using PRICE [81], which utilizes a seed-and-extend assembly strategy, and aligned back to the reference to identify spliced junctions. We ran PRICE for 20 cycles using the anchored read pairs (those which map uniquely near the gene retrocopy insertion site) as the seed sequences.

### Simulation of novel gene retrocopy insertions

Retrogene insertions were simulated by adding insertions of spliced, polyadenylated mRNA transcripts to sample TCGA-60-2711-11 (LUSC-2711 Normal) using bamsurgeon [82]. Bamsurgeon can add structural variants (including insertions) to existing BAM files through local assembly followed by modification of the assembled contig, simulation of paired read coverage (100 paired end base pairs with 300 unsequenced insert base pairs), realignment, and replacement into the original BAM. We added a total of 2,000 insertions from 200 different processed mRNAs (Table S11 in Additional file 1) to LUSC-2711, and downsampled the resultant BAM from 60× average coverage to 40×, 30×, 20×, 10×, and 5× using DownSampleSam, part of the Picard suite of utilities [83]. We used GRIPper to detect the spiked-in processed mRNAs to evaluate the detection characteristics. At 60× coverage we obtained perfect precision and a recall of 0.751 (1,501 true positives and 499 false negatives with no false positives). As expected, recall decreases with decreasing coverage (Table S10 in Additional file 1). In general, false negatives are due to single exon genes (for example, *OR7G2*) at high coverage and mainly due to insufficient read support at low coverage. Since we combined reads from both tumor and normal genomes for all TCGA samples in this study, which have coverage of 30× or greater, detection of germline insertions was done on samples with an effective coverage of 60× or greater.

### Identifying gene retrocopy insertions included in the reference genome assembly

GRIPs in the reference genome that are not present in other individuals will appear as deletions relative to the reference. To detect these, we cross-referenced the deletion data from the 1,000 Genomes Project [34,57] with pseudogene annotations from GENCODE/ENCODE [84] and Yale [1]. Deletions were obtained in variant call format from the 1,000 Genomes Project FTP server, and pseudogene annotations where obtained from the UCSC Genome Browser [85], and from pseudogene.org human build 65 [3]. To allow for repetitive sequences in gene UTRs we allowed the deletion to span a region up to three times larger than the surrounded pseudogene annotation. We also required homology between the deleted sequence and the source gene of the annotated pseudogene. A list of the GRIPs ascertained in this way is included in Additional file 1 (Table S7 in Additional file 1), two of which correspond to both of the processed pseudogene deletion polymorphisms (pseudocopies of *GCSH *and *ITGB1*) mentioned in a previous study [17].

### Strategy for low-pass genome sequence data and tumor/normal pairs

In order to ascertain insertion sites from a large collection of genomes sequenced at low (2× to 5×) coverage, or to ensure maximum sensitivity in ascertaining cancer-specific insertions, we combine data across multiple samples. This is accomplished simply by extracting discordant reads where one end maps to an exon and the other end elsewhere in the reference genome from each genome of interest, and analyzing the merged set of discordant reads *en masse *while keeping track of the sample identifier associated with each discordant pair of mapped reads. When insertions are called, all genomes contributing reads to a call are considered to have the insertion.

### Calculating coverage of gene annotations

In order to test for enrichment or depletion of gene retrocopy insertions relative to gene annotations, we must have an accurate figure for how much of the reference genome assembly is covered by the set of annotations used. For both human and mouse, we used UCSC genes [86]: human version 5 and mouse version 5. From BED formatted versions of these annotation tracks, the bedCoverage tool from the Kent source utilities was used to calculate the fraction of the genome covered. To calculate enrichment, we performed a one-sample proportions test with continuity correction using the prop.test function in R [87].

### Calculating distance between GRIP profiles

The Jaccard distance [52] is defined as:

(1) $$J_{\delta }\left(A,B\right)=\frac{\left|A\cup B|-|A\cap B\right|}{\left|A\cup B\right|}$$

where *A *and *B *are sets of gene retrocopy insertions for two genomes.

### Estimating the rate of gene retrocopy insertion

Given a parameter *θ *and an effective population size *N_e_*, we can calculate the per-generation mutation rate *μ *by [49]:

(2) $$\theta =4N_{e}\mu$$

where θ is estimated by:

(3) $${\hat{\theta }}_{W}=\frac{S}{\overset{n-1}{\underset{i=1}{\sum }}\frac{1}{i}}=\frac{S}{a_{n}}$$

where S is the number of segregating sites and *n *is the number of individuals. Since we have *n *= 1,024 and *S *= 48, *a_n _*= 7.508, and ${\hat{\theta }}_{W}=48/7.508=6.394$. If we assume an effective population size of 10,000, *μ *= 6.394/40,000 ≈ 1/6,256 GRIPs per individual per generation. Including the 10 pseudogenes present in the reference but deleted in one or more individuals in the 1,000 Genomes Project data (Table S7 in Additional file 1), which likely indicate GRIPs that are included in the reference, our estimate for *θ *becomes ${\hat{\theta }}_{W}=58/7.508=7.725$ yielding a rate of *μ *= 7.725/40,000 ≈ 1/5,178 gene retrocopy insertions per individual per generation.

This estimate requires the segregating sites to be neutral markers. We determined that, on the whole, GRIPs qualify as neutral markers with Tajima's D test, based on commonly used critical values of -2.0 and +2.0 corresponding to purifying and diversifying selection, respectively [58]:

(4) $$D=\frac{{\hat{\theta }}_{T}-{\hat{\theta }}_{W}}{\sqrt{\hat{V}}}$$

where ${\hat{\theta }}_{T}$ is the mean number of differences between any two individuals in terms of the chosen segregating sites. In this case:

(5) $$D=\frac{3.412-6.394}{\begin{array}{c} \sqrt{9.073} \end{array}}\approx -0.990$$

### Data access

Data from the 1,000 Genomes Project [34] is available from the website [88]; Table S1 in Additional file 1 contains a list of individual genomes downloaded for analysis as part of this study. Data from The Cancer Genome Atlas is available to authorized users through the Cancer Genomics Hub [89]; a list of tumor/normal pairs used in this analysis is included as Table S3 in Additional file 1. The genomes of 17 inbred mouse strains [47] are available through the Wellcome Trust Sanger Institute Mouse Genomes Project [90]. The genomes of ten individual chimpanzees [54] are available through the PanMap project [91].

## Abbreviations

AML: acute myeloid leukemia; bp: base pair; ASW: African ancestry in south-west US; BRCA: breast cancer; CEU: Utah residents with northern and western European ancestry; CHB: Han Chinese in Beijing, China; CHS: Han Chinese in southern China; CLM: Colombian in Medellin, Colombia; CNV: copy number variant; COAD: colorectal adenocarcinoma; FDR: false discovery rate; FIN: Finnish from Finland; GBM: glioblastoma multiforme; GBR: British from England and Scotland; GO: gene ontology; GRIP: gene retrocopy insertion polymorphism; JPT: Japanese in Tokyo, Japan; LINE: long interspersed element; LUAD: lung adenocarcinoma; LUSC: lung squamous carcinoma; LWK: Luhya in Webuye, Kenya; miRNA: microRNA; MXL: Mexican ancestry in Los Angeles; ORF: open reading frame; OV: ovarian carcinoma; PCR: polymerase chain reaction; PUR: Puerto Rican in Puerto Rico; siRNA: small interfering RNA; TCGA: The Cancer Genome Atlas; TSI: Toscani in Italy; UTR: untranslated region; YRI: Yoruba in Ibadan, Nigeria

## Competing interests

The authors declare that they have no competing interests.

## Authors' contributions

AE and DH conceived the study, AE and TB developed software and performed the analysis, AE wrote the paper, DE produced visualizations, LD, CH, RW, and members of the Broad Institute produced sequencing data and performed primary sequence alignments. All authors read and approved the final manuscript.

## Supplementary Material

Additional file 1 **See Supplemental Data (Additional file **3**) for individual table and column descriptions**. Click here for file

Additional file 2 **Contains splice junctions detected in inserted sequences**. The FASTA-formatted file can be opened with any text editor. Click here for file

Additional file 3 **Contains descriptions of supplemental tables (Additional file **1**), supplemental figures, and sequences**. Click here for file

Additional file 4 **Archival version of GRIPper**. We recommend downloading the latest version from github [78]. Click here for file
