# Supplementary material for: Retrotransposition of gene transcripts leads to structural variation in mammalian genomes
Source: Genome Biol. 2013 Mar 13;14(3):R22. doi: 10.1186/gb-2013-14-3-r22 (PMC3663115; doi:10.1186/gb-2013-14-3-r22)
Supplement: Additional file 2 — Contains splice junctions detected in inserted sequences. The FASTA-formatted file can be opened with any text editor. [file gb-2013-14-3-r22-S2.PDF]

Supplemental Figures for *Retrotransposition of gene transcripts leads to structural variation in mammalian genomes*

1 Supplemental Table Descriptions

Table S1: Samples names for 1000 Genomes data. List of sample identifiers used in the 1000 genomes portion of this study.

Table S2: Insertions from 1000 Genomes data. Locations of GRIPs are indicated by "chr" (chromosome), "start", and "end" coordinates. The inferred orientation is indicated in the "strand" column. The identity of the progenitor gene is indicated in the "Gene" column. The number of exons mapped with discordant reads relative to the parent gene is indicated in the "Exons" column. The "Reads" column denotes the total number of supporting reads across all samples (the number of samples is indicated in the "Genomes" column). If a GRIP is located in an intron or exon of another gene annotation this is noted in the "Host Gene" column. If a GRIP is located in a repeatmasker annotation (generally highly diverged to allow unique mapping), this is indicated in the "RepeatMasker" column. The number of populations covered by the individuals where a GRIP was detected (see "ID List") is indicated in the "Populations" column. For each population, the fraction of population members with a given GRIP is listed in the "Population Frequency" column.

Table S3: Sample names for TCGA tumor/normal pairs. This table shows the correspondence between sample names assigned by The Cancer Genome Atlas and abbreviated sample names used in this study.

Table S4: Insertions from TCGA Genomes. Column names and meanings are a subset of those listed for Table S2.

Table S5: Insertions from TCGA and 1000 Genomes combined. Where column names match those for Table S2 the description is the same. The "Description" column adds a brief description for each gene in the "Gene" column. If a more precise location than the range given by "Start" and "End" can be provided, it is given here. This may be slightly outside the "Start" and "End" range in some cases due to clipped reads. The "EN Site" column indicates the predicted endonuclease cleavage site (see Figure S5). Where target site duplications were detected, "TSD" indicates the sequence. The number of breakpoints detectable from soft-clipped read mappings is indicated in the "Breakpoints" column. If a "Y" is present in the "Exon Junc" column, this indicates one or more exon-exon junctions was detectable from the read mappings. The sequences of 5' and 3' junctions are indicated in the corresponding columns. Cancer-specific insertions are indicated under the heading "Cancer-specific retrogene insertions".

Table S6: GRIPs in 17 inbred mouse strains relative to C57BL/6J assembly mm9/NCBI m37.

Column descriptions that correspond to those in Table S2 are identical with the exception that IDs ("ID List") refer to individuals representative of the indicated inbred strain.

Table S7: Pseudogene deletion polymorphisms relative to hg19/GRCh37. GRIPs present in hg19 and absent from one or more individuals in the 1000 Genomes Project.

Table S8: Functional classification of GRIP progenitor genes. The "Symbol" column contains the HUGO gene symbol associated with a given progenitor gene annotation. The curated functional annotation for each gene is noted in the "General Function" column.

Table S9: Insertions from PanMap chimpanzee genomes. GRIPs collected from an analysis of 10 chimpanzee genomes. Column descriptions are taken from Table S2.

Table S10: GRIP simulation results to evaluate the performance of processed pseudogene detection on simulated data. As described in the methods section, a total of 2000 insertions spanning 200 different source genes were added to a BAM file for sample TCGA-60-2711-11 and detected with GRIPper. At each selected coverage level (indicated in the "Coverage" column), "TP" indicates the number of true positives, "FP" indicates the number of false positives, and "FN" indicates the number of false negatives. Precision and recall are calculated from the TP, FP, and FN columns.

Table S11: Gene-wise detection of simulated GRIPs in a 60x alignment. Related to the 60x coverage row in Table S10, this table shows the number of true positives (TP) and false negatives (FN) for each of the 200 genes spiked-in to TCGA-60-2711-11.

Table S12: Read counts spanning GRIP Breakpoints from TCGA samples. The first three columns indicate the breakpoint location and identity and subsequent columns indicate how many aligned reads in the combined tumor and normal BAM files span the breakpoint in the patient indicated by the column header. Fields marked "DEL" indicate a deletion of > 200 bp is present at the junction in the corresponding individual in both the tumor and normal samples. In order to span the junction location indicated in the "Position" column, a read must have at least 20bp aligned to the reference on either side of the junction and must not be soft-clipped.

2 Somatic gene retrocopy insertions in tumor samples

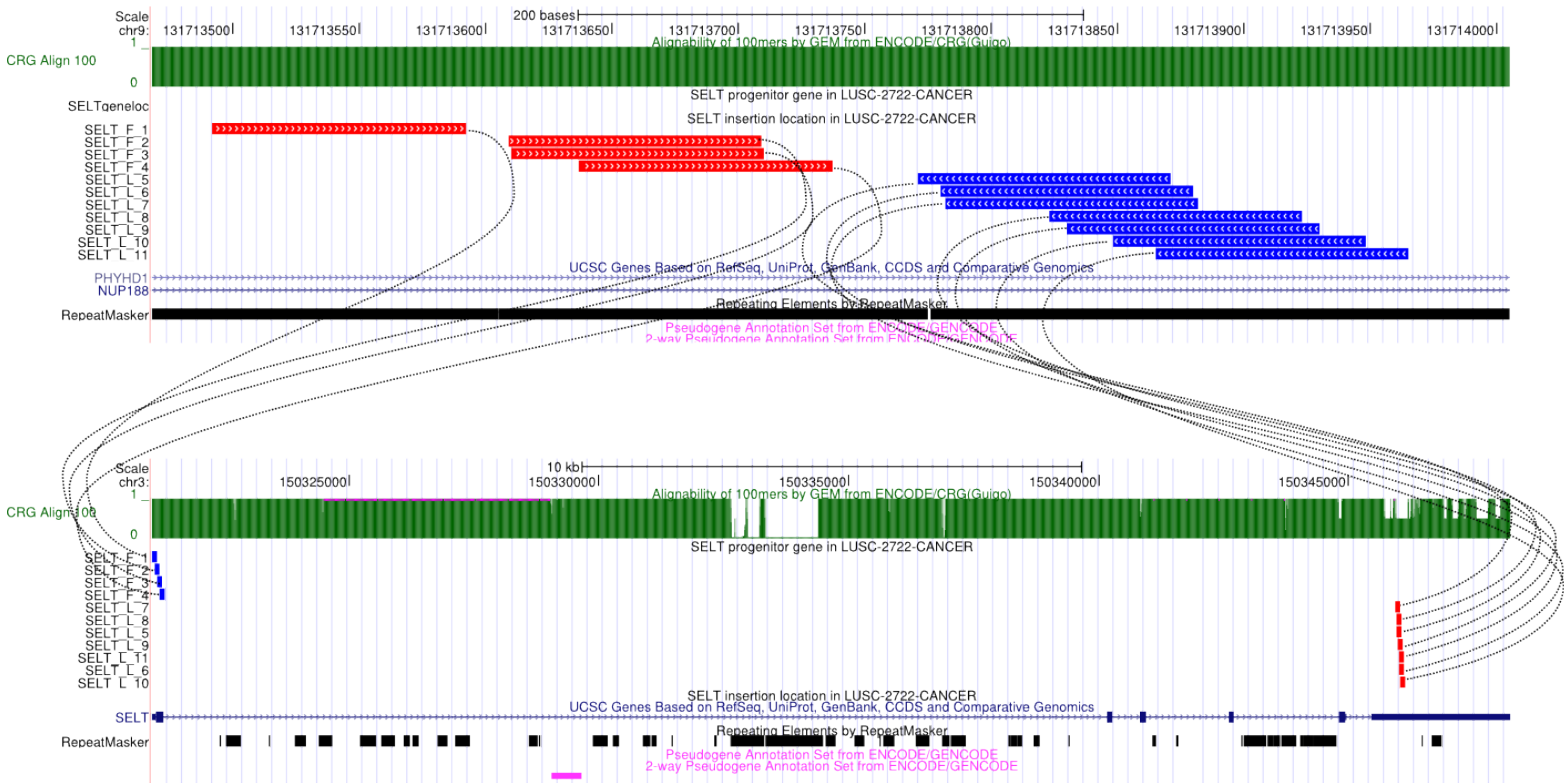

Supplementary Figure 1: Insertion of a *SELT* (Selenoprotein T precursor) transcript in lung squamous cell tumor sample TCGA-60-2722-01, not present in the corresponding cancer sample. Read mapping locations are shown in two images from the UCSC Genome Browser, those mapping to the insertion site on chr9 on top, and those mapping to the the source gene on the bottom. Reads are colored according to the strand to which they map: red corresponds to the top strand and blue corresponds to the bottom. Lines between reads indicate the pairing between discordant reads. The green bar (CRG Align 100) represents mapability of 100bp reads. Note that the insertion occurs in an intron of the *PHYHD1* gene.

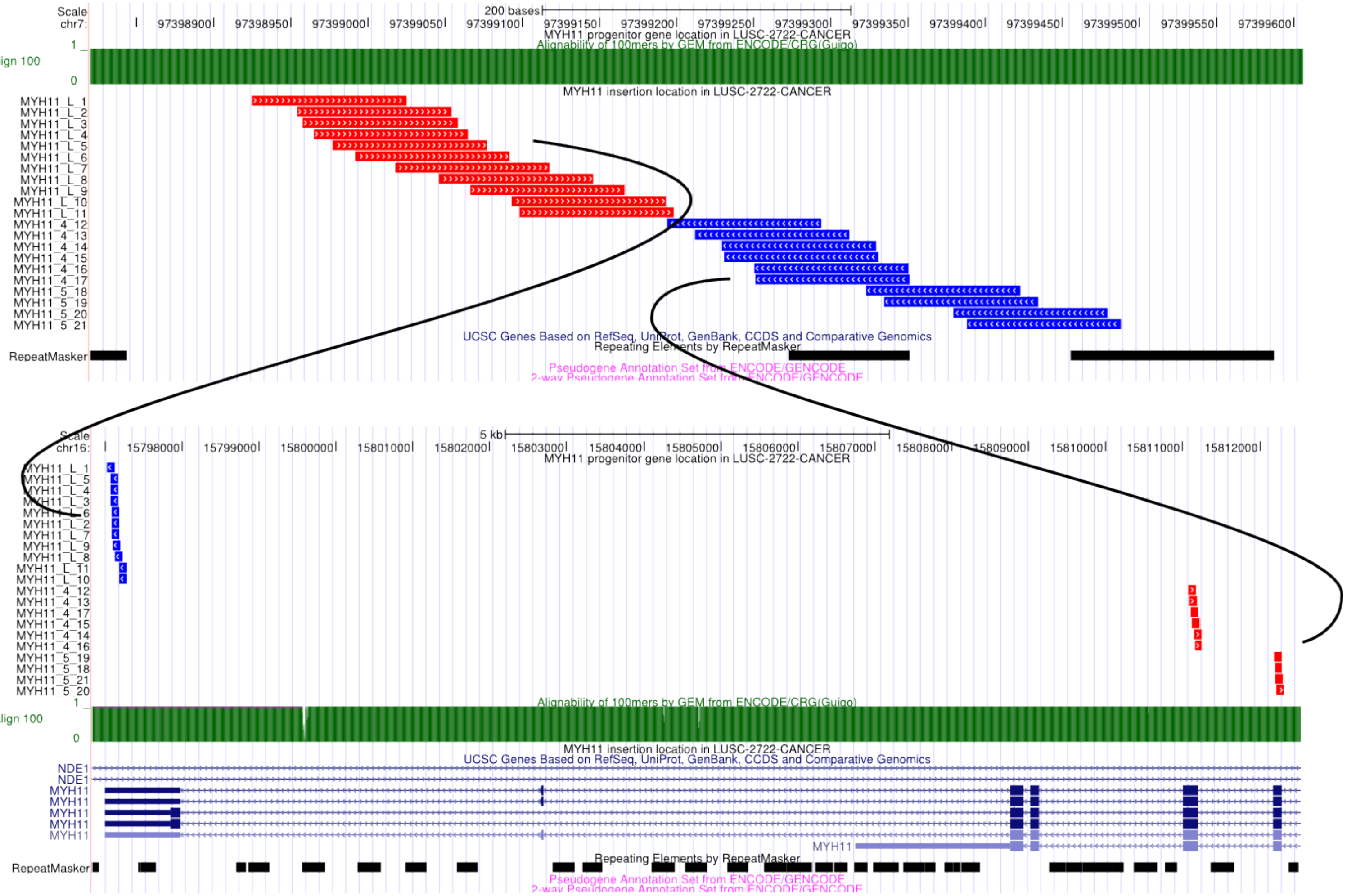

Supplementary Figure 2: Insertion of a *MYH11* (Smooth muscle myosin heavy chain 11) transcript in lung squamous cell tumor sample TCGA-60-2722-01, not present in the corresponding cancer sample. Read mapping locations are shown in two images from the UCSC Genome Browser, those mapping to the insertion site on chr9 on top, and those mapping to the the source gene on the bottom. Reads are colored according to the strand to which they map: red corresponds to the top strand and blue corresponds to the bottom. Lines between reads indicate the pairing between groups of discordant reads. The green bar (CRG Align 100) represents mapability of 100bp reads.

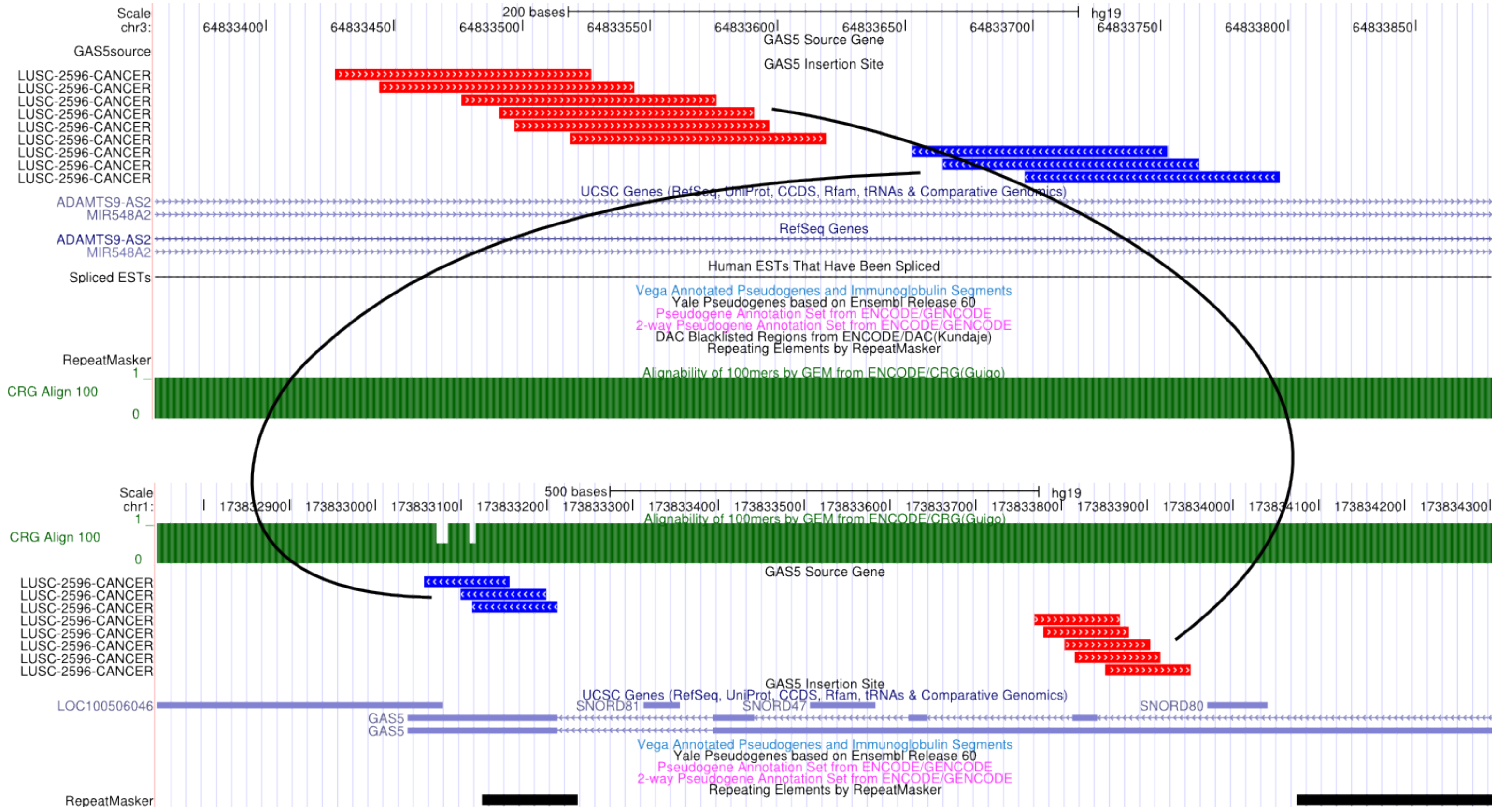

Supplementary Figure 3: Insertion of *GAS5* (Growth arrest-specific 5 non-coding RNA) transcript in lung squamous cell tumor sample TCGA-34-2596-01, not present in the corresponding cancer sample. Read mapping locations are shown in two images from the UCSC Genome Browser, those mapping to the insertion site on chr9 on top, and those mapping to the the source gene on the bottom. Reads are colored according to the strand to which they map: red corresponds to the top strand and blue corresponds to the bottom. Lines between reads indicate the pairing between groups of discordant reads. The green bar (CRG Align 100) represents mapability of 100bp reads. Note that the insertion occurs in an intron of the *ADAMTS9-AS2* non-coding RNA, which may also overlap an intron of *MIR548A2*, a pri-miRNA.

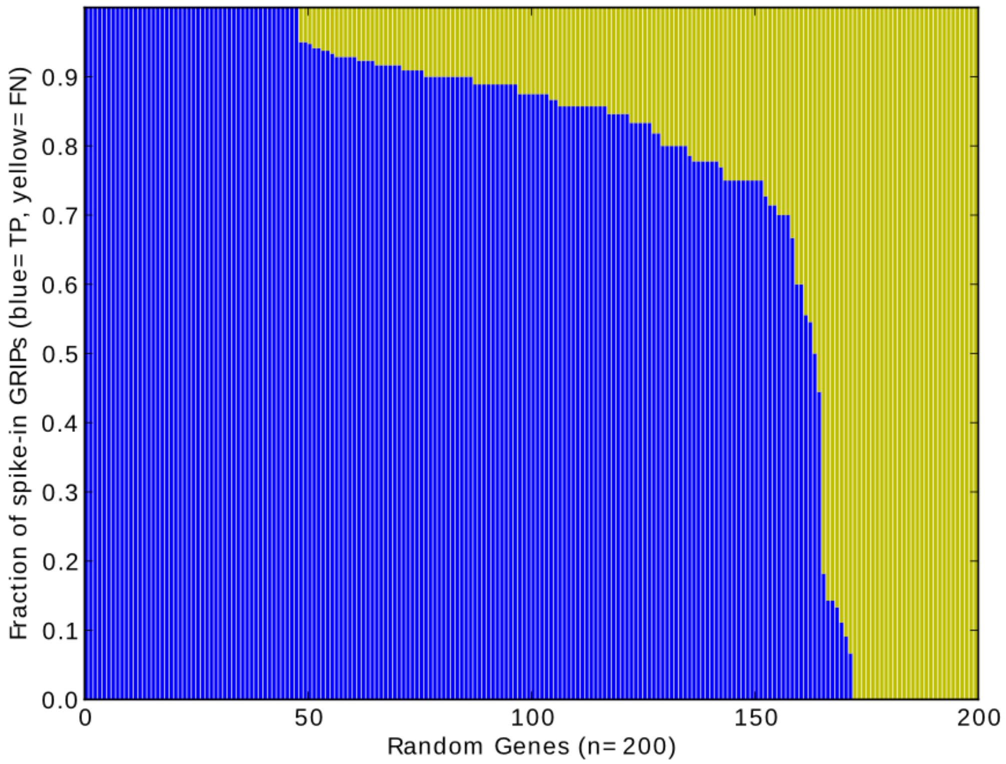

Supplementary Figure 4: Simulation of gene retrocopy insertions from 200 source genes: 2000 insertions total over 200 genes. Insertions were spiked in to sample TCGA-60-2711-11 (LUSC-2711, 60x coverage) at 50% minor allele frequency. Insertions were detected with GRIPper, true positive (TP, blue) and false negative (FN, yellow) rates for each gene are shown. See Table S11 for TP and FN counts for each gene.

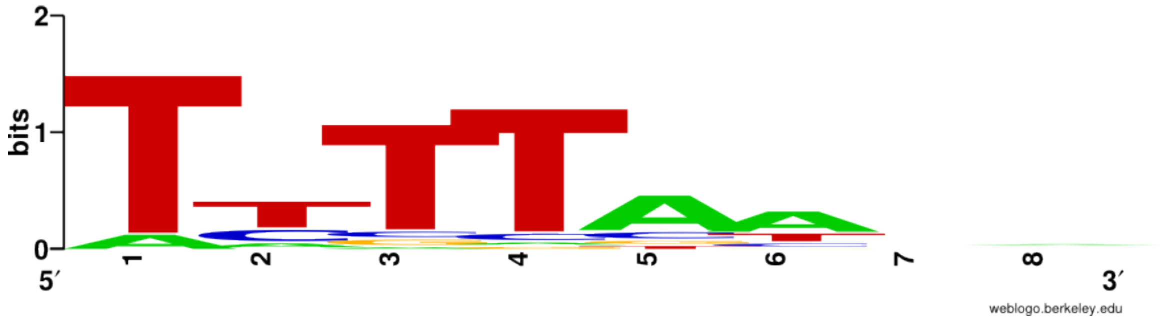

Supplementary Figure 5: Consensus endonuclease cleavage site derived from 3' junctions (Table S5).

3 Sequences of exon-exon junctions

See supplementary file Splice_junctions.fa
